# Comprehensive treatment of von Hippel‐Lindau disease: A case report

**DOI:** 10.1002/cai2.94

**Published:** 2023-09-20

**Authors:** Xuesong Li, Zheng Mo, Zhuo Yu

**Affiliations:** ^1^ School of Clinical Medicine Tsinghua University Beijing China; ^2^ Department of Oncology Beijing Tsinghua Changgung Hospital Beijing China

**Keywords:** sorafenib, surgery, tislelizumab, treatment, von Hippel‐Lindau disease

## Abstract

von Hippel‐Lindau (VHL) disease is a rare autosomal dominant multiorgan disease characterized by several benign and malignant tumors rich in vascular, as well as cysts in other organs. A great clinical treatment strategy is significantly warranted for good prognosis of patients with VHL disease. Herein, we reported a case of a 45‐year‐old woman diagnosed with VHL disease with spinal hemangioblastoma (HB) and clear cell renal cell carcinoma (ccRCC). Four years after the resection of the right kidney, a recurrent RCC in the right kidney and a malignant lesion in the left kidney were observed. This patient was started on sorafenib (800 mg, daily) and tislelizumab (200 mg per 3 weeks). After 6 months of treatment, the size of renal cell carcinoma was dramatically reduced and renal function improved. More importantly, she achieved partial response during the whole treatment. Microscopically, intramedullary masses resection was done and the HB in T4‐5 thoracic spinal was removed. Neurologic symptoms such as numbness and pain were remarkably alleviated. Additionally, tislelizumab‐induced elevation in liver transaminase levels and hypothyroidism were revered by hepatoprotector and levothyroxine, respectively. In short, comprehensive treatment strategies may benefit patients with VHL disease, especially with HB and ccRCC.

## Graphical abstract

Mechanisms of therapeutic strategies for von Hippel‐Lindau (VHL) disease are discussed. Sorafenib and tislelizumab exhibited the ability of anti‐VHL disease by targeting mutation of VHL‐induced oncogenes expression including vascular endothelial growth factor receptors, platelet‐derived growth factor receptors, and blocking PD‐1/PD‐L1 pathway.

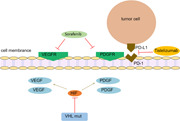

**Abbreviations**
ACT: adoptive T cell therapy
ccRCC: clear cell renal cell carcinoma
CT: computed tomography
FDA: Food and Drug Administration
HB: hemangioblastoma
ICIs: immune‐checkpoint inhibitors
MRI: magnetic resonance imaging
PDGF: platelet‐derived growth factor
PDGFR: platelet‐derived growth factor receptor
PD‐1: programmed cell death protein 1
PD‐L1: programmed cell death ligand 1
PR: partial response
RA: retinal angioma
TILs: tumor‐infiltrating lymphocytes
Trm‐like: tissue‐resident memory‐like
VEGF: vascular endothelial growth factor
VEGFR: growth factor receptor
VHL: von Hippel‐Lindau

## 1 INTRODUCTION

von Hippel‐Lindau (VHL) disease is a rare autosomal dominantly inherited disease. Mutant *VHL* loses the ability to degrade downstream factor HIF, resulting in the activation of pro‐tumor signaling pathways and the occurrence of disease [1]. Multisystemic disorders, including central nervous system hemangioblastoma (HB), retinal angioma (RA), clear cell renal cell carcinoma (ccRCC), and pancreatic tumors occur in VHL patients.

Sorafenib, an oral multiple tyrosine kinases inhibitor, can suppress the proliferation of tumor cells by targeting vascular endothelial growth factor receptor (VEGFR) and platelet‐derived growth factor receptor (PDGFR) [2]. Mutation of the *VHL* gene can lead to constitutive activation of HIF downstream targets, including vascular endothelial growth factor (VEGF) and platelet‐derived growth factor (PDGF), which could enhance the progression of tumors. Belzutifan, the first HIF‐2α inhibitor, was approved for VHL disease‐associated RCC and central nervous system HB by the Food and Drug Administration (FDA) in 2021 [3], but it is not available in China at present. The programmed cell death protein 1 (PD‐1)/programmed cell death ligand 1 (PD‐L1) pathway plays a vital role in cancer immune escape and is viewed as a potential target for cancer therapy. A multicenter study conducted in China demonstrated that tislelizumab, a PD‐1 inhibitor, exhibited potent antitumor activity consistent with other PD‐1 inhibitors and well tolerated in RCC patients [4].

A recent study reported that deletion of *VHL* in CD8+T cells induced the differentiation of tissue‐resident memory‐like (Trm‐like) tumor‐infiltrating lymphocytes (TILs) in an HIF‐dependent manner, and anti‐PD‐1 treatment in combination with *VHL*‐deficient CD8+ adoptive T cell therapy (ACT) reduced tumor burden and increased survival, which can provide protection from tumor challenge [5]. More importantly, a comparative study uncovered that there was a significant correlation between positive PD‐L1 expression and aggressive pathological features in VHL‐associated ccRCC [6]. In addition, it has been demonstrated that drugs targeting the VHL/HIF/VEGF signaling pathway, such as sorafenib can dramatically improve the outcome of patients with advanced ccRCC [7].

Herein, we report a case of a 45‐year‐old woman with a diagnosis of VHL with ccRCC, central nervous system HB who was treated with a series of comprehensive treatments.

## 2 CASE REPORT

**Figure 1:**
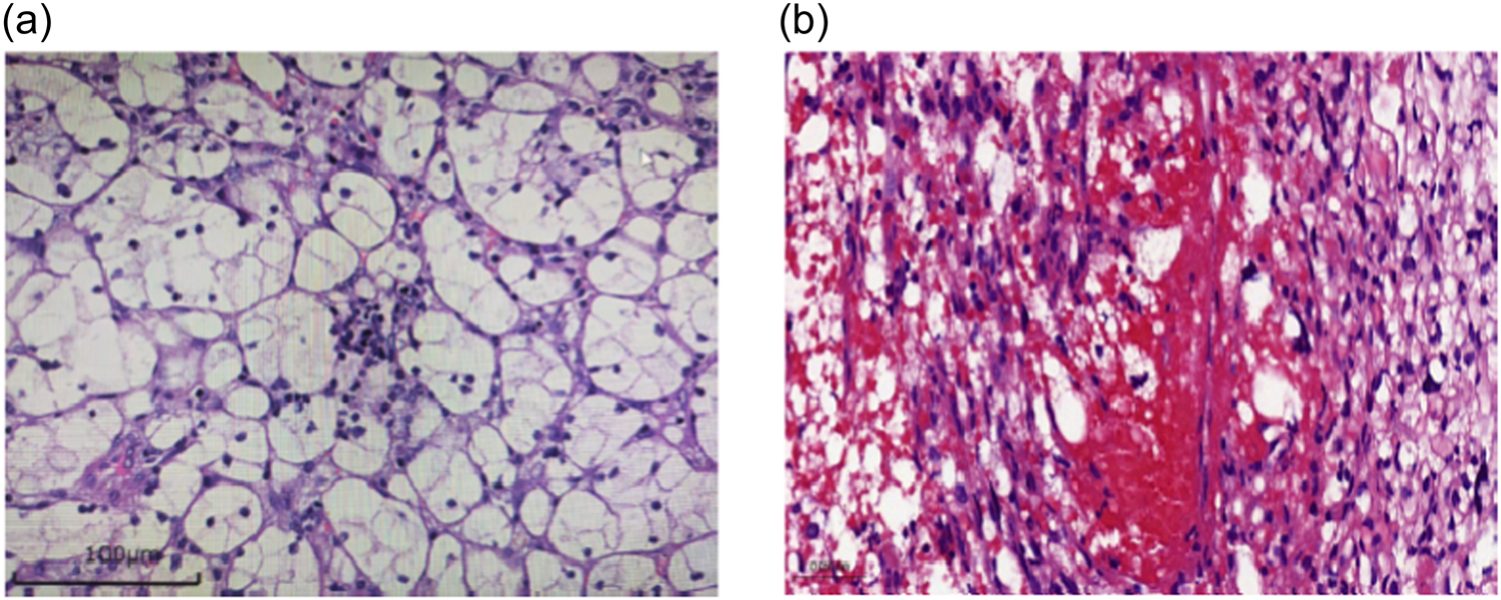
Histology of tumor tissues. (a) ccRCC in right kidney (magnification, ×400). (b) HB in T4‐5 thoracic spinal (magnification, ×400). ccRCC, clear cell renal cell carcinoma; HB, hemangioblastoma.

A 45‐year‐old woman was admitted to the hospital with complaints of intermittent dizziness on October 21, 2018. A neurological examination indicated a power of 5/5 and normal tone in all limbs. She denied suffering from any chronic or infectious disease. There was no family history. Magnetic resonance imaging (MRI) found an HB of the left cerebellar. Meanwhile, computed tomography (CT) unraveled a malignant lesion in the right kidney. She had to undergo resection of cerebellar HB and radical resection of the right kidney with the histological diagnosis of grade G2 by Fuhrman ccRCC (Figure 1a). Moreover, *VHL* germline mutation analysis showed *VHL* exon 2–3 deletion.

**Figure 2:**
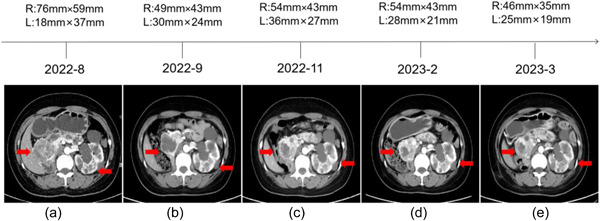
Response of VHL‐RCC to treatment. The change in the sizes of a recurrent ccRCC in the right kidney and a malignant space‐occupying in the left kidney during the whole treatment (a–e). RCC, renal cell carcinoma; VHL, von Hippel‐Lindau.

**Figure 3:**
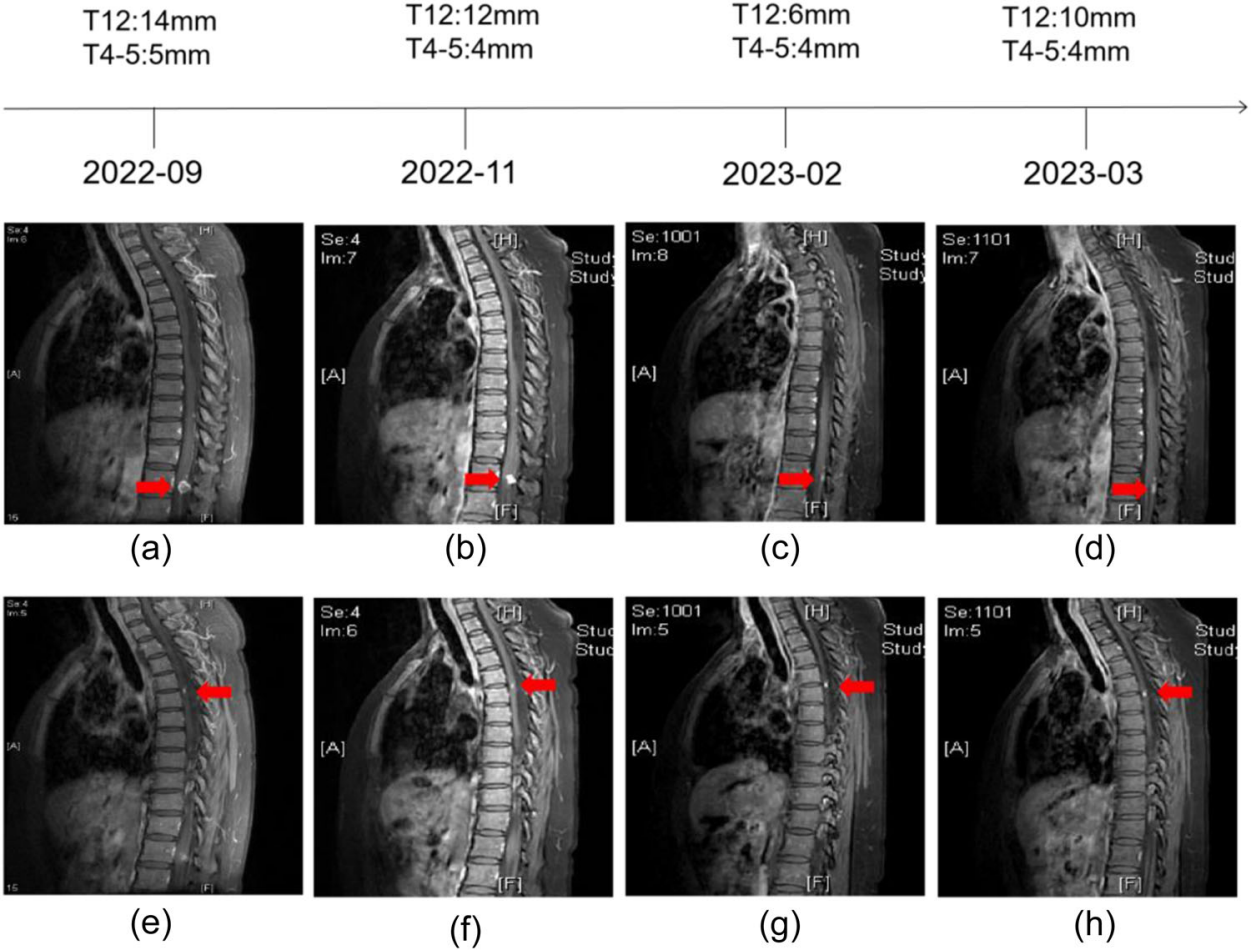
Spinal MRI of the patient during the whole treatment. The changes in the sizes of T4‐5 and T12 spinal HB during the whole treatment (a–h) can be observed. MRI, magnetic resonance imaging.

She visited our hospital for a check‐up on August 3, 2022. The abdominal CT revealed a recurrent ccRCC in the right kidney and a malignant lesion in the left kidney (Figure 2a). This patient was started on sorafenib (800 mg, daily) for treatment. On September 1, 2022, MRI showed a T4‐5 and a T12 thoracic spinal HB, respectively (Figure 3a,e). The abdominal CT revealed that both the RCC in the left kidney and the recurrent ccRCC in the right kidney were significantly reduced (Figure 2b). This patient achieved partial response (PR). Later, on November 4, 2022, abdominal CT showed that the size of both the RCC in the left kidney and the recurrent ccRCC in the right kidney were slightly increased (Figure 2c), but this patient still managed to achieve PR. Considering the enlarging trend of tumors, we gave her tislelizumab intravenously (200 mg per 3 weeks) from November 18, 2022. In addition, MRI exhibited that the size of T12 thoracic spinal HB was significantly decreased while the size of T4‐5 thoracic spinal HB was stable (Figure 3b,f). On February 17, 2023, compound glycyrrhizin tablets (50 mg tid) were prescribed to ameliorate the patient's hepatic injury. Abdominal CT revealed that this patient continuously achieved a PR (Figure 2d). The size of T12 thoracic spinal HB was decreased while the size of T4‐5 thoracic spinal HB remained unchanged (Figure 3c,g). On March 21, 2023, she presented to our clinic with complaints of weakness, aversion to coldness, and numbness in the lower legs. Diagnosis of hypothyroidism was confirmed with thyroid function. Meanwhile, she received oral 25 μg per day of Euthyrox. Both the RCC in the left kidney and the recurrent ccRCC in the right kidney were reduced (Figure 2e). However, the size of T4‐5 thoracic spinal HB was still unchanged, which might contribute to numbness of her lower legs (Figure 3d,h). Microscopically, intramedullary masses resection was done and the HB in T4‐5 thoracic spinal removed (Figure 1b). After a 2‐ week follow‐up, the liver function enzymes went back to normal, and thyroid function improved to some extent, which exhibited a continuous trend in improvement (Table 1).

**Table 1:** Change of liver, renal, and thyroid functions during the whole treatment.

	2022–8	2022–9	2022–11	2023–2	2023–3	2023–4
Cre (41–73 μmol/L)	159.0	137.0	134.0	128.0	123.0	116.0
eGFR (mL/min/1.73 m^2^)	32.8	39.0	40.1	42.4	44.5	47.7
ALT (7–40 U/L)	9.1	15.6	20.0	73.4	42.5	23.1
AST (13–35 U/L)	9.7	17.5	17.9	42.2	34.5	23.9
FT3 (3.1–6.8 pmol/L)	−	−	−	6.34	2.29	2.6
FT4 (12–22 pmol/L)	−	−	−	26.5	7.4	10.7
TSH (0.27–4.20 μIU/mL)	−	−	−	0.1	72.6	36.7
TPO‐Ab (<34 KIU/L)	−	−	−	28.3	93.5	−
Tg‐Ab (0–115 KIU/L)	−	−	−	88.1	121.0	−

## 3 DISCUSSION

Sorafenib is a first‐line drug for metastatic ccRCC, but its efficacy for relapsed VHL‐associated ccRCC is rarely reported. In a Phase II study, the organ‐specific response rate was 52% in RCC and 4% in central nervous system HB after pazopanib for 24 weeks in 31 VHL patients [8]. In addition, sunitinib treatment for four cycles in VHL patients showed a significant response in RCC [9]. In this case, the patient developed a recurrence of RCC after resection of the right kidney. After treatment with sorafenib combined with tislelizumab, the size of recurrent ccRCC was significantly reduced, and the size of the RCC in the left kidney did not significantly increase.

Liver injury and hypothyroidism following tislelizumab treatment were observed in this patient. According to present guidelines, immune‐checkpoint inhibitors (ICIs) such as PD‐1 inhibitors should be suspended when liver transaminase levels increase 3–5 times the upper limit of normal in patients and be perpetually abandoned when liver transaminase levels exceed this threshold value [10]. This patient presented with isolated elevation in liver transaminase levels and liver function gradually returned to normal after hepatoprotector treatment. Besides, studies also suggested that patients with ICI treatment who present hypothyroidism should receive levothyroxine substitution after precluding the possibility of adrenal insufficiency. So, replacement therapy with Euthyrox was initiated for this patient, and her thyroid function improved.

## 4 CONCLUSION

VHL disease is a systemic condition referring to multiorgans and comprehensive treatment, including target therapy, immunological therapy, and surgical therapy may be an effective and safe treatment choice for VHL patients, especially with HB and ccRCC.

## AUTHOR CONTRIBUTIONS

**Xuesong Li**: Conceptualization (equal); data curation (equal); formal analysis (equal); investigation (equal); methodology (equal); software (equal); writing—original draft (equal). **Zheng Mo**: Supervision (supporting). **Zhuo Yu**: Funding acquisition (equal); resources (equal); writing—review & editing (equal).

## CONFLICT OF INTEREST STATEMENT

Professor Zhuo Yu is a member of the *Cancer Innovation* Editorial Board. To minimize bias, he was excluded from all editorial decision‐making related to the acceptance of this article for publication. The remaining authors declare no conflict of interest.

## ETHICS STATEMENT

This article is a practice‐oriented case study description that made extensive use of secondary information sources and also drew upon the professional knowledge of the co‐authors. As such, the creation of this case study article neither involved any formal research study nor human participation. Moreover, an IRB review was not required for this article.

## INFORMED CONSENT

Written informed consent was required from the patient for the publication of this article following the explanation of the purpose of the manuscript.

## Data Availability

The data that support the findings of this study are available from the corresponding author upon reasonable request.
